# Arriving at Results Efficiently: Using the Enhanced Evaluability Assessment Approach

**DOI:** 10.5888/pcd12.150413

**Published:** 2015-12-24

**Authors:** Jan L. Losby, Marla Vaughan, Rachel Davis, Aisha Tucker-Brown

**Affiliations:** Author Affiliations: Marla Vaughan, Rachel Davis, Aisha Tucker-Brown, Division for Heart Disease and Stroke Prevention, Centers for Disease Control and Prevention, Atlanta, Georgia.

## Abstract

Evidence, particularly practice-based evidence, is needed to guide public health practice. With the goal of contributing to practice-based evidence, the Division for Heart Disease and Stroke Prevention at the Centers for Disease Control and Prevention combined and streamlined aspects of an evaluability assessment and an effectiveness evaluation to create the Enhanced Evaluability Assessment (EEA). This approach offers a viable and less costly alternative to evaluators and practitioners by quickly identifying and evaluating models with evidence of effectiveness that can be replicated and expanded. The EEA can be applied to a range of public health topics, not just cardiovascular health. This article provides a step-by-step description of the EEA.

## Introduction

Public health agencies often face substantial fiscal constraints and challenges to identifying and implementing strategies that have the greatest effect on improving health. Given this reality, there is an increased need for efficient methods to identify programs or interventions with demonstrated evidence of effectiveness (1) that can be replicated and expanded. Three categories of evidence can guide public health practice, one of which is practice-based evidence (2). Practice-based evidence can be obtained by conducting formal evaluation of practices being implemented by public health practitioners (3) in a natural setting.

With the goal of contributing to practice-based evidence, the Division for Heart Disease and Stroke Prevention (DHDSP) at the Centers for Disease Control and Prevention (CDC) used a staged evaluation approach — a pre-evaluation assessment to appraise a program’s capacity and readiness for an effectiveness evaluation and then an effectiveness evaluation of the selected site. This staged approach guides subsequent investment of resources in evaluating public health practices and serves as a platform for developing strong evaluation designs for programs in the field, thereby yielding practice-based evidence.

We describe a newly created evaluation approach, the Enhanced Evaluability Assessment (EEA), which incorporates key aspects of the staged approach while streamlining and truncating the process to provide a quicker assessment of readiness, an effectiveness evaluation, and dissemination of practice-based evidence. This approach may improve informed decision making and lead to enhanced evaluation and implementation of public health programs. This approach can be applied to all areas of public health inquiry, not simply cardiovascular health. We describe each step of the EEA approach, including considerations and lessons learned.

## Expanding Practice-Based Evidence

Since 2008, DHDSP has used pre-evaluation assessments — the Systematic Screening and Assessment (SSA) and the Evaluability Assessment (EA) — to determine whether a program demonstrates potential promise to address cardiovascular disease outcomes. The SSA verifies that the intervention is 1) fully implemented, 2) operating as intended, 3) operating consistently over time, 4) capable of collecting and extracting data on outcomes, and 5) achieving its desired effects, according to the data (2). Leviton and Gutman (4) assert that the SSA process is an efficient method of sifting through many promising interventions, and the EA process developed by Wholey (5) provides information through methods such as site visits and document review to identify whether a program is evaluable. Both of these methods stop short of rigorously evaluating the program to assess effectiveness. For DHDSP, the SSA and EA lasted approximately 12 months. Once a program was selected and deemed evaluable and ready for an effectiveness evaluation, a 30-month effectiveness evaluation was launched to determine the intervention’s implementation effectiveness relative to improved health outcomes. The effectiveness evaluation was a rigorous process that included interviews and quantitative data abstraction and analyses to describe the core components of the program, determine changes in health outcomes, and provide recommendations for replication.

Although this multi-staged approach was fruitful, the timeframe of approximately 42 months was often deemed too long, especially given the shortage of evidence, limited resources, and a pressing need for public health action. There is considerable interest among public health practitioners and stakeholders to arrive at results more quickly without sacrificing scientific rigor. Several methodologic advances have been made that move rapid evaluation approaches from shortcuts to legitimate approaches (6), such as rapid assessments (7), rapid-cycle evaluation (8), and rapid-feedback evaluations (9). DHDSP asked questions to determine whether there was a viable alternative and what would be needed for a new method. These questions were:

- **How much information is enough?** When implementing the initial SSA and effectiveness evaluation model, it was clear that the SSA did not yield enough information to determine effectiveness or guide replication in the field, which were determined to be the most important necessities of adding to practice-based evidence. Therefore, collecting information to meet these goals was prioritized when developing a new method.
- **How do we identify evaluable programs quickly and efficiently?** Because DHDSP works with many partners, the idea of using these partnerships to both gain an understanding of areas of need related to practice-based evidence and identify potentially evaluable programs was explored. This action would shorten the time spent during the SSA nomination process substantially.
- **What is an acceptable level of rigor?** When conducting the SSA, EA, and effectiveness evaluation together, evaluators were able to collect data over an extended period. Therefore, evaluators could provide technical assistance to sites to help with data quality, account for programmatic changes, and collect data at different points to identify changes in outcomes over the project period. With a shorter timeline, the sites would need to have quality, retrospective data available to provide to evaluators.

After brainstorming answers to these questions, DHDSP developed the framework for the EEA (Figure). Using this method, DHDSP would identify interventions ready for evaluation, describe the core components, and show the effectiveness of innovative interventions to increase replication while meeting the needs of internal and external stakeholders to have an efficient and shorter timeframe.

**Figure:**
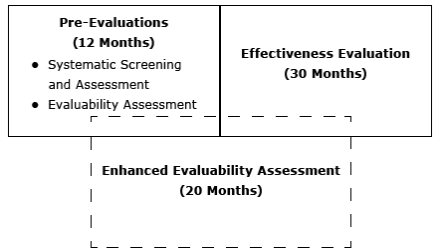
Conceptual model of the Enhanced Evaluability Assessment.

## Key Differences Between the EEA and the SSA, EA, and Effectiveness Evaluations

The EEA brings together key features of the SSA, EA, and effectiveness evaluation methods, with key modifications.

**Identification of evaluable programs.** The identification of evaluable programs had previously occurred through an SSA nomination process (4). The SSA call for nominations was open to state and local health departments, health care providers, and other entities working to reduce cardiovascular disease, whether receiving CDC funding or not. Under the EEA, the review of promising practices in the field is done by in-house staff only. The pool of programs includes state and local health departments or health care providers — programs either funded by CDC or simply known to CDC through partners or from previous assessments. Once identified, prospective programs were contacted to discern their fit for an effectiveness evaluation. The EEA still incorporates the SSA method of using a set of criteria but eliminates the sometimes long process (ie, 3–6 months) of collecting program nominations and then coordinating and conducting an external panel review to assess the nominations. Although this streamlined approach makes the selection process more efficient, the pool of potential nominated programs may be more limited than if there were a self-nomination process from the field. However, after careful consideration, it was determined that this portion of the assessment process could be shortened with minimum sacrifice given the broad networks in-house staff have.

**Program description.** Creating a comprehensive program description, including an accurate logic model, is an important part of the EEA process. This component is similar to the EA process except that it takes less time (3 months compared with 6 months), because the effort relies on telephone calls and sharing existing documents rather than making a site visit.

**Effectiveness evaluation.** The EEA uses a mixed-methods evaluation approach, similar to the previously used method, but relies more on existing data and a shorter timeframe than the 30-month effectiveness evaluation.

**Expert panel.** Under the SSA and EA, expert panels are instrumental in 1) the initial selection of the site to undergo an EA and 2) determining the site’s suitability for evaluation after the EA. Expert panels include people with diverse yet relevant backgrounds and experiences to ensure that multiple perspectives are represented. DHDSP selects subject matter experts from clinical, academic, state, and local practice settings; CDC; and other federal agencies. The EEA uses an internal expert panel at the end of the evaluation period to determine the intervention’s public health impact and the quality of the evidence (1).

## Step-by-Step Description of the EEA Approach

The EEA has several steps that start with the selection of the program and end with an expert panel review followed by dissemination. The Box provides the timeframe for the 20-month project.

**Box. Steps of the Enhanced Evaluability Assessment**

**Table:** 

Step	Month Number(s)
Selection of program to evaluate	1–3
Evaluation questions and evaluation design	3–6
Data collection	6–16
Data analysis	17
Preliminary results shared with program	18
Report writing	18–19
Final briefing with program	19
Expert panel convened	20
Dissemination	20

**1. Selection of program to evaluate.** A review of promising practices in the field is done by in-house CDC staff members. DHDSP relies on CDC project officers, evaluation liaisons, or other CDC staff who work with programs and have strong ties to state and national public health networks. A list of potential programs, including both CDC-funded and non–CDC-funded, are assessed by using a set of criteria (ie, potential impact, health effect size, reach, feasibility, sustainability, transferability, and data capacity). Once the list is compiled, prospective programs are contacted by CDC to discern their fit for an effectiveness evaluation. The point of contact and key staff at the potential site are asked to provide relevant program documents and verification of access to data sources needed for the evaluation. It is essential that the selected program have access to at least 6 months of data, that these data are accessible to the program, and that the intervention itself is well-established. The EEA approach can be applied to a range of program interventions. The intervention drives the need for the type of data; for example, the data could be health outcome data or behavior change outcomes.

The selected program is involved in the entire EEA process and has regular (usually twice monthly) meetings or calls with the evaluators to discuss progress and provide input into the project. Having a champion and single point of contact at the site is critical to successfully conducting the EEA and obtaining the requested data in a short timeframe. It is estimated that site staff engagement in the EEA process is approximately 6 hours per week. This level of engagement is slightly less than the anticipated engagement for an effectiveness evaluation, which is approximately 10 hours per week.

**2. Evaluation questions and evaluation design.** The selected site is substantially involved in the evaluation planning and implementation process. Once the site has agreed to participate in the EEA, the next step is to engage the selected site in an in-depth discussion of their evaluation needs and determine a mutually satisfying focus for the EEA. It is important that the evaluation be value-added for the site, because little compensation is being given to the selected site to participate in the evaluation.

**3. Data collection.** Data for the EEA are collected through document review, site visit, interviews, and quantitative data abstraction. The evaluators obtain existing program documents, reports, and intervention descriptions to prepare a comprehensive program description including an accurate logic model. This information is obtained by working with a point of contact at the site of the selected program. After a thorough review of this information, a site visit is scheduled.

The purpose of the site visit is to conduct interviews of key personnel and partners involved in program implementation. Interviews and focus groups are conducted as needed, and site visitors are given an opportunity to observe the program firsthand. This is also the time when the evaluators can get clarification on any lingering questions that arise during the earlier review of written materials. In addition to the document review and site visit, a quantitative data abstraction is completed.

Evaluators work with the program to abstract relevant data to be used as evidence of promise or effectiveness in the strategies being implemented. In many cases, a modest stipend is given to the selected program to secure the requested health outcome or implementation data. Once the initial program description is completed, based on the document review, site visit, and other data collection, the information is shared with the program to determine whether there are any clarifications or misunderstandings that need to be resolved.

**4. Data analysis.** The data collection for an EEA is a rigorous process that uses both quantitative and qualitative methods. Therefore, the analysis portion of the study includes a review of documents and identification of themes and a statistical analysis of the quantitative data collected. The analyses vary based on the needs of the study but always include triangulating the data and gaining consensus of all reviewers.

**5. Preliminary results shared with program.** A key discussion point occurs with the program after the analysis of process and outcome data. These preliminary findings are presented to the program, usually through a conference call or in webinar format, so that they can be reviewed carefully and discussed as a group. Any potential contradictions or areas that need follow up or clarification are identified through this step before final reports are written and findings are further disseminated. Any changes are then incorporated into the reports.

**6. Report writing.** Two reports are written that summarize the findings of the evaluation. The first report, the program description, is usually written midway through the evaluation project and provides a detailed description of the program and key findings from the qualitative data collection. This report includes the goals and expected outcomes of the program, the core components of the program, factors affecting program implementation, and the logic model of the program. The second report incorporates much of the program description report but focuses on the final results of all data collection and analyses. Specific elements of the report include a brief executive summary that can be used as a stand-alone document, a background section, an overview of the study design and methods, results, and a conclusion with recommendations. The overview of the evaluation design and methods describes the components, design, and evaluation questions; outlines the qualitative and quantitative methods; and addresses study and data limitations. The bulk of this report is the results section. The results begin with information from the program description report, then a discussion of implementation effectiveness, practice and program related outcomes (including program reach), and program effectiveness. Program effectiveness results generally include health system outcomes and clinical and patient-level outcomes of interest in the evaluation. The conclusion provides a discussion of the findings and considerations for replication. The considerations for replication also link to the original selection criteria so that the program can be assessed by an expert panel using those criteria.

**7. Final briefing with program.** A formal final briefing is provided to the program in webinar format. The presentation includes an overview of the evaluation methods, process and outcome evaluation findings, final conclusions, and reflections on the evaluation. Staff from the program who were not directly involved in the evaluation study are invited to participate to share thoughts related to the evaluation methods, findings, and future implications. Clarifications and refinements are made to the final report based on this discussion.

**8. Expert panel convened.** A unique aspect of the EEA is the use of an expert panel at the end of the evaluation to assess the intervention using a CDC-created conceptual framework (1). Two components of the framework are public health impact and quality of the evidence. Public health impact consists of 5 elements — effectiveness, reach, feasibility, sustainability, and transferability. Quality of evidence consists of 4 levels — weak, moderate, strong, and rigorous. Experts receive a scoring matrix that has key concepts defined and has questions to inform the assessment process. Individuals with senior-level expertise in the areas of evaluation, chronic disease public health, or best practices are identified and invited to participate on the expert panel. Invitees may be internal or external to CDC. The invitation for participation includes a statement on the level of commitment (a total of 8 hours to review the materials, make the assessment, and participate in the convening call or meeting), the estimated timeframe for reviewing the materials and completing the scoring process, and a tentative date for convening the panel in a small-group discussion format (in-person or virtual). A statement related to compensation can be included if panel members are external to CDC or do not represent other federal agencies.

To prepare the panel for the small-group discussion, a description of the intervention, outcomes, literature related to the intervention, instructions for reviewing materials, and a scoring sheet are emailed in advance for submitting scoring sheets to CDC. Scores are tallied, and overall comments are compiled. Summaries are shared during the small group discussion in PowerPoint presentation format. The goal of convening the small group is to provide panel members the opportunity to discuss assessments, engage in dialogue, and explore content related to the level of evidence based on the public health impact determination and quality of evidence provided. A CDC representative convenes the panel, facilitates the discussion, and clarifies points as needed. Input from the expert panel is incorporated into the final evaluation documents.

**9. Dissemination.** In collaboration with the program, findings from the evaluation are shared internally with the program and CDC and shared externally on the websites of both the program and CDC. Implementation guides, field notes, or other briefing documents may also be created to enable replication by other programs. Whenever possible, the evaluation findings are also disseminated through peer-reviewed journals and professional conferences.

## Conclusion

To build practice-based evidence, appropriately designed evaluation methods must be implemented with rigor and efficiency. As a new methodology, the EEA incorporates these essential qualities by collecting primary and secondary data while reducing costs and time. With one EEA completed and 2 others under way, early indications suggest that this approach offers a viable and less costly alternative to evaluators and practitioners by quickly identifying and evaluating models with evidence of effectiveness that can be replicated and expanded.

As with every methodology, there are some challenges and lessons learned that should be considered when determining whether to implement an EEA versus other evaluation approaches. As noted, the pool of nominations may be smaller than the broader call for nominations used by the SSA, which may inadvertently omit some innovative or promising practices. Also, an approach with a broader call for nominations may be more appropriate when the focus area is new or emerging or when the evaluator does not have access to networks to identify potentially promising programs. It is also essential with the EEA that the evaluators establish a strong relationship with the site at the beginning of the process for the evaluation to be successful. It is important to consider the evaluation needs of the program and to offer feedback and reports that are useful to the program for program improvement, building evaluation capacity, and demonstrating successes in ways that are relevant for their stakeholders. DHDSP has found this to be a key incentive for programs’ participation and ongoing engagement. Another lesson learned is that it is critical during the initial discussions with potential programs to understand what data are collected or are accessible and how these data are managed and extracted. Using the expert panel at the end of the evaluation is a unique aspect of the EEA. Because DHDSP is using criteria from the CDC framework, it is necessary for the summaries of the findings to speak clearly to these criteria or it is difficult for the panel to assess during the scoring process. Depending on the needs of the evaluation and the focus of the program’s intervention, evaluators may wish to explore other frameworks or assessment criteria for the expert panel to use.

Keeping these early lessons learned in mind will help evaluators determine whether the EEA is a viable evaluation approach for their work. Highlighting this approach is intended to increase evaluation capacity, support informed decision making, and lead to improved evaluation and implementation of public health programs. Because the EEA is responsive to stakeholder needs and enables the timely dissemination of evaluation findings, the EEA has the potential to have broad application for a range of public health topics.
